# The trend and the disease prediction of vascular endothelial growth factor and placenta growth factor in nontuberculous mycobacterial lung disease

**DOI:** 10.1038/srep37266

**Published:** 2016-11-23

**Authors:** Chou-Han Lin, Chin-Chung Shu, Chia-Lin Hsu, Shih-Lung Cheng, Jann-Yuan Wang, Chong-Jen Yu, Li-Na Lee

**Affiliations:** 1 Department of Internal Medicine, Far-Eastern Memorial Hospital, New Taipei City, Taiwan; 2 Graduate Institute of Clinical Medicine, College of Medicine, National Taiwan University, Taipei, Taiwan; 3 Department of Traumatology, National Taiwan University Hospital, Taipei, Taiwan; 4 Department of Internal Medicine, National Taiwan University Hospital, Taipei, Taiwan; 5 Department of Chemical Engineering and Materials Science, Yuan Ze University, Zhongli City, Taoyuan County, Taiwan; 6 Department of Laboratory Medicine, National Taiwan University Hospital, Taipei, Taiwan

## Abstract

Nontuberculous mycobacteria (NTM)-lung disease (LD) is an increasing health problem worldwide. The diagnosis of this disease remains difficult, however the application of placenta growth factor (PlGF) and vascular endothelial growth factor (VEGF) has not yet been studied. We screened patients with *Mycobacterium avium* complex or *M. abscessus* isolated from sputum, and enrolled 32 patients with NTM-LD and 93 with NTM pulmonary colonization. The NTM-LD group had a lower body mass index, higher proportion of bronchiectasis, more respiratory symptoms and pulmonary lesions, and higher titers of sputum acid-fast stain than the NTM pulmonary colonization group. The plasma level of PlGF was lower in the NTM-LD group than in the NTM colonization group, whereas the level of VEGF was higher in the NTM-LD group. In multivariable logistic regression analysis excluding NTM cultures, the predictive model for NTM-LD included sputum AFS titer, a nodular-bronchiectasis radiographic pattern, plasma VEGF/PlGF ratio, and chest radiographic score (VEGF/P1GF ratio became not significant as a factor in multivariable generalized linear model). The four-factor predictive index had good positive likelihood ratio and negative likelihood ratio for predicting NTM-LD in the patients with NTM in their sputum.

The incidence of nontuberculous mycobacteria (NTM)-lung disease (LD) has been reported to be increased even in subjects without human immunodeficiency virus infection^1^,^2^,^3^,^4^, and it has become a health problem worldwide^5^,^6^. The American Thoracic Society (ATS) guidelines^5^ require multiple criteria to diagnose NTM-LD, including clinical and radiographic findings and microbiology, including two or more sets of sputum positive samples for the same NTM infection within 1 year. This process takes time and is inefficient because cultures are time-consuming^7^.

In addition, the clinical relevance of NTM growth in a sputum culture is much less than 100%, because NTM exists ubiquitously in the environment. The relevance of *Mycobacterium avium* complex (MAC) in a sputum culture has been reported to be only 35–42%^8^,^9^, and 33% for *M. abscessus* (MAB)^9^. Making an early diagnosis and treatment are important because NTM-LD can be fatal in critically ill patients and in those who do not receive appropriate treatment^10^,^11^,^12^. Thus, predictors for the clinical diagnosis of NTM-LD are needed, especially when waiting for confirmatory mycobacterial cultures.

Microbiological findings of smear positivity and NTM species have been reported to be predictors for NTM-LD^9^, in addition to a radiographic bronchiectasis pattern^13^. Due to low specificity with a single parameter, combining these factors with biomarkers may be more useful. However, systemic inflammatory molecules in the blood such as interferon-gamma and C-reactive protein have been reported to be poorly associated with making a diagnosis, indicating that host cellular immunity may not be adequately responding to a NTM infection^14^. Local pulmonary biomarkers may be better predictors. Of these markers, vascular endothelial growth factor (VEGF) is responsible for angiogenesis in granulomatous inflammation and is produced by macrophages to induce an immune recruitment response when mycobacteria enter the airway^15^,^16^. Higher levels of VEGF have been reported in patients with pulmonary *Mycobacteria* infections^17^,^18^,^19^. Placenta growth factor (PlGF) is produced from bronchial epithelial cells and is a biomarker that shares significant sequence homology with VEGF at an amino acid level. It can inhibit proliferation, promote cell death, and it has been reported to potentially represent local inflammation^20^,^21^,^22^. Angiogenesis of granuloma and local responses from macrophages and bronchia epithelial cells play important roles in NTM-LD, however VEGF and PlGF have rarely been investigated. The aim of this prospective study, therefore, was to investigate dynamic changes in PlGF and VEGF between NTM-LD and pulmonary colonization, and analyze the potential diagnostic value of these biomarkers.

## Materials and Methods

### Patient enrollment

This prospective study was conducted at National Taiwan University Hospital from October 2012 to September 2015. The research ethics committee of National Taiwan University Hospital approved the study, which was conducted in accordance with the approved guidelines. All of he enrolled participants provided written informed consent.

Patients aged ≥20 years who had respiratory sample(s) culture-positive for MAC or MAB in the past 12 months were identified, as these two species are most commonly responsible for NTM-LD^1^,^23^,^24^. Patients were recruited consecutively when they visited our chest or infection clinic and were diagnosed with NTM-LD or NTM pulmonary colonization based on the ATS diagnostic guideline^5^. Briefly, NTM-LD was diagnosed if all of the following were met: (1) two or more sputum culture-positive specimens for the same NTM species; (2) chest images (radiography or computed tomography [CT]) demonstrating lesions compatible with NTM-LD (i.e., fibro-cavitary lesions or multi-focal bronchiectasis; (3) presence of respiratory symptoms; and (4) no obvious alternative diagnosis at that time.

A patient with a positive sputum culture for MAC or MAB but who did not fulfill all of these criteria was defined as having MAC or MAB pulmonary colonization. We enrolled a control group from July 2015 to June 2016 in the same study hospital with the same number of participants as those with NTM-LD. All of the control group had negative chest radiographic images or were sputum-negative for NTM. Patients were excluded if they had human immuno-deficiency virus infection, had received anti-NTM treatment for more than 1 week, or had acute illnesses which caused systemic inflammatory response syndrome.

### PlGF and VEGF measurements

Peripheral blood samples were obtained from all participants using heparinized tubes. Plasma was immediately collected and stored at −20 °C. The samples were examined in random order by a technician blinded to the patient’s clinical status. Plasma PlGF and VEGF were measured using an enzyme immunoassay kit (R&D Systems, MN, USA) according to the manufacturer’s instructions.

### Data collection

Clinical data, including age, sex, body mass index (BMI), smoking status, co-morbidities, prior pulmonary tuberculosis (TB), symptoms, laboratory data, and radiographic findings upon enrollment were recorded in a standardized case report form with default selection. Current smokers were defined as those who had smoked >100 cigarettes, with the last time of smoking within 1 month prior to the study^25^.

Symptoms were reviewed according to the patients’ history. Cough ≥3 weeks was defined as chronic cough. Symptom duration was defined as the period prior to the date of the first confirmed positive culture before enrollment. Co-morbidities and prior TB were recorded from medical records. A chest specialist and a radiologist interpreted the chest images. The extent of lung lesions was scored as in a previous study (details in the Supplement File)^26^,^27^, and radiographic patterns of the main pulmonary lesions were categorized as fibro-cavitary, nodular-bronchiectasis, and others. Sputum-positive samples for acid fast bacilli in fluorescent staining were then confirmed by Ziehl Neelsen stain. Acid fast smears (AFS) were interpreted as 0, scanty, 1, 2, 3, and 4^28^. Mycobacterial cultures were performed as previously described^10^.

### Statistical analysis

Inter-group differences were analyzed using the Student’s *t* test for numerical variables and the *chi*-square test for categorical variables. Relative risks were analyzed using a generalized linear model with Poisson regression. Multivariable logistic regression was used to identify factors associated with NTM-LD in the patients who were sputum-positive for NTM. In stepwise forward variable selection, all potential predictors were included. Statistical significance was set at a two-sided *p* value of less than 0.05.

The discriminative power of each significant predictor between those with NTM-LD and colonization was compared using receiver operating characteristic (ROC) curve analysis, and the area under the curve (AUC). Optimal values were calculated using the Youden index. All analyses were performed using SPSS software version 19.0 (SPSS, Chicago, IL).

## Results

**Figure 1:**
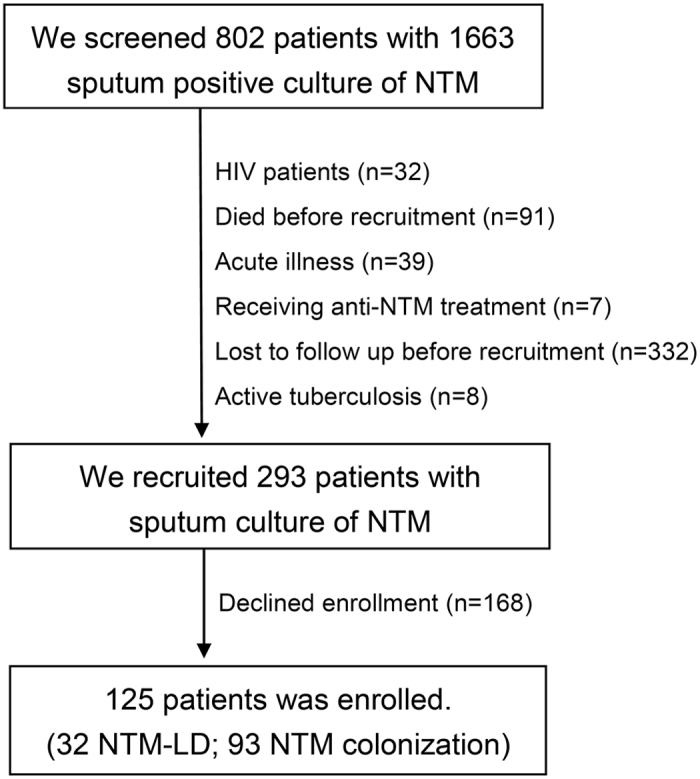
Flow chart of screening and enrolling participants with sputum positive for nontuberculous mycobacteria (NTM). Because enrolling period and parent population of the control group are not the same as participants with NTM in sputum. We did not describe the control group in the flow chart. LD, lung disease.

Thirty-two patients with NTM-LD (19 MAC and 13 MAB) and 93 with NTM pulmonary colonization (68 MAC and 25 MAB) during the study period were enrolled (Fig. 1). All of the patients in the colonization group had ≥2 sputum samples cultured for mycobacterium except for one. Thirty-two participants were also enrolled in the control group. The mean age and number of male patients in each group were similar (NTM-LD group: 63 years, 41% male; NTM colonization group: 67.4 years, 44%; and control group: 59.8 years, 41%) (Table 1). In terms of clinical characteristics, the NTM-LD group had lower BMI, higher rate of bronchiectasis, and more symptoms, especially chronic cough, than those with NTM pulmonary colonization. Other underlying co-morbidities and smoking status were comparable.

Regarding radiographic findings, 74% of the participants had chest CT, with more in the NTM-LD group (91% vs. 68%, *p* = 0.011). In the patients with NTM-LD, typical fibro-cavitary and nodular-bronchiectasis patterns accounted for 31% and 53% of the findings, respectively, compared to 0%and 33% in those with NTM colonization. The extent of pulmonary lesions by radiographic score (maximum score 18) was higher in the NTM-LD group (mean ± standard deviation: 3.5 ± 1.6) than in the colonization group (1.9 ± 2.0).

Respiratory specimens were AFB-positive in 21 (66%) and 13 (14%) of the NTM-LD and NTM colonization groups, respectively (*p* < 0.001). Among them, 13 with NTM-LD and 3 with NTM pulmonary colonization had high-grade (3+ ~ 4+) AFS positivity, with average AFS titers of 1.8 vs. 0.2 (*p* < 0.001), and 2.5 vs. 0.2 (*p* < 0.001) AFS-positive specimens per patient. Mycobacterial cultures for the same NTM in the NTM-LD group were positive in 4.1 sets, compared to 1.3 sets of the NTM colonization group (*p* < 0.001).

**Figure 2:**
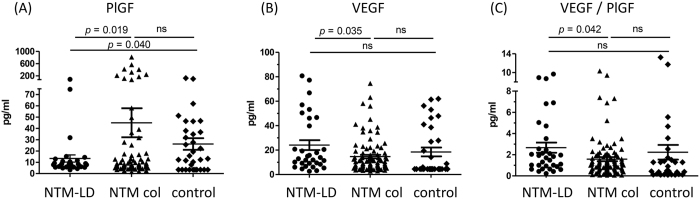
Plasma levels of **(A)** placenta growth factor (PlGF), **(B)** vascular endothelial growth factor (VEGF), and **(C)** their ratio according to nontuberculous mycobacteria (NTM)-lung disease (LD), pulmonary colonization (col), and control group. The cross lines are mean value and error bars are standard error of the mean. ns, not statistically significant.

The plasma level of PlGF was higher in the NTM colonization group than in the NTM-LD group (45.0 vs. 13.4 pg/ml, *p* = 0.019), and the PlGF level in the NTM-LD group was lower than in the control group (13.4 vs. 26.18 pg/ml, *p* = 0.040) (Fig. 2). However, the plasma level of VEGF was higher in the NTM-LD group than in the NTM pulmonary colonization group (24.1 vs. 14.7 pg/ml, *p* = 0.035). The ratio of VEGF divided by PlGF was higher in the NTM-LD group than in the colonization group (2.7 vs. 1.6, *p* = 0.042).

In terms of clinical presentations, PlGF was correlated with dyspnea (Pearson correlation: 0.249, *p* = 0.05) and underlying asthma (Pearson correlation: 0.508, *p* < 0.001). There was no significant difference in PlGF level between those with and without COPD (84 ± 258.1 vs. 40.9 ± 116.7 pg/ml, *p* = 0.443). VEGF was positively correlated with old age, lower BMI, underlying malignancy, and high number of cultures positive for NTM (Pearson correlation: 0.209, −0.202, 0.188, and 0.211, *p* = 0.019, 0.024, 0.036 and 0.018, respectively). The AFS-positive grade and radiographic score had borderline significant correlations with VEGF (Pearson correlation: 0.167 and 0.153, *p* = 0.063 and 0.091, respectively).

The relative risks for the outcome of NTM-LD according to PlGF, VEGF, and the ratio of VEGF/PlGF using the generalized linear model were 0.994 (95% CI: 0.987–0.999, p = 0.049), 1.108 (95% CI: 1.007–1.029, p = 0.001), and 1.136 (95% CI: 1.007–1.280, p = 0.038), respectively. There was a linear relationship between the biomarkers and NTM-LD. We performed multivariable logistic regression analysis for predictors of NTM-LD in the patients who were sputum-positive for NTM. Factors entered into the regression analysis included age, sex, NTM species, and all significant factors in univariable analysis, including BMI, radiographic pattern and score, chronic cough, AFS titer, mycobacterial species, plasma PlGF, plasma VEGF, and VEGF/PlGF ratio. Because more than two sets of positive NTM sputum cultures were required according to the diagnostic criteria, and because that data could not be obtained at baseline, the number of positive NTM cultures was not included in the regression model. In the final model by forward factor selection (Table 2), the AFS titer in the sputum (odds ratio [OR]: 2.007, 95% CI: 1.181–3.411), nodular-bronchiectasis radiographic pattern (OR: 8.580, 95% CI: 2.069–35.575), and plasma VEGF/PlGF ratio (OR: 1.372, 95% CI: 1.068–1.761) were independent predictors of NTM-LD. Radiographic score had borderline significance (OR: 1.272, 95% CI: 0.964–1.680). Multivariable analysis using the generalized linear model with Poisson regression was performed (Table 3), and the relative risks for NTM-LD were 1.636 (1.295–2.068) by sputum AFS titer (per 1 grade increment), 1.189 (1.010–1.399) by radiographic score (per 1 score increment), 1.048 (0.923–1.189) by VEGF/PlGF ratio (per 1 unit increment), and 2.212 (1.049–4.651) by the presence of a nodular-bronchiectasis pattern. The relative risks were smaller than odds ratios for the same factors.

**Figure 3:**
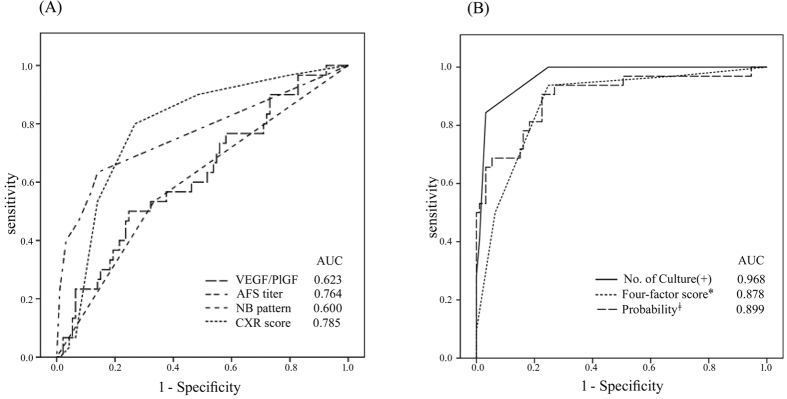
Receiver operating characteristic (ROC) curves for predicting nontuberculous mycobacteria (NTM)-lung disease in patients with sputum positive for NTM by **(A)** the four significant factors in the multivariable regression and by **(B)** the numbers of sputum culture positive for NTM, the probability (ǂ) from the multivariable logistc regression analysis, and the predictive index score (*) by the four factors. The four factors using for the predictive index score were ratio of plasma vascular endothelial growth factor (VEGF)/placenta growth factor (PlGF) >1.8, positive sputum acid fast smear (AFS), radiographic pattern of nodular-bronchiectasis (NB) pattern, and radiographic (chest X film, CXR) score >2. One point was given for each factor and the sum was the predictive index score.

ROC curve analysis for NTM-LD among the patients with NTM in their sputum (Fig. 3) revealed AUC values of 0.764 (95% CI: 0.650–0.875) for AFS titer in the sputum, 0.785 (95% CI: 0.688–0.873) for radiographic score, 0.623 (95% CI: 0.511–0.741) for VEGF/PlGF ratio, and 0.600 (95% CI: 0.481–0.719) for a nodular-bronchiectasis pattern, all of which were lower than that for the number of sputum cultures positive for NTM (AUC = 0.968, 95% CI: 0.941–0.995). Using the probability developed from the multivariable regression model (AUC = 0.899, 95% CI: 0.828–0.969), the predicting AUC value was similar to that by the number of sputum cultures positive for NTM (Fig. 3B). The probability equation is described in the Supplement File.

Using the Youden index to identify the optimal cut-off values of continuous independent factors, we defined one point each for sputum AFS titer >0, radiographic score >2, VEGF/PlGF ratio >1.8, or the presence of a nodular-bronchiectasis pattern. The predictive index from this four-factor model was then obtained with a range from 0 to 4. The AUC for NTM-LD with this four-factor model (Fig. 3B) was 0.878 (95% CI: 0.809–0.947), which was similar to that with the probability from the regression model. If the VEGF/PlGF ratio was excluded, the AUC decreased to 0.857 (95% CI: 0.785–0.929) with the remaining three factors and was inferior to the AUC with the number of sputum-positive cultures for NTM.

We then compared the performance of different combinations of the predictors (Table 4). The predictive index with a four-factor model score >2 had 50% sensitivity, 94% specificity, 8.3 positive likelihood ratio (LR), and 0.53 negative LR, compared to 94% sensitivity, 75% specificity, 3.76 positive LR, and 0.08 negative LR for a score >1.

## Discussion

In the present study, the plasma level of VEGF was higher in the patients with NTM-LD than in those with NTM pulmonary colonization, while the plasma PlGF level had an opposite trend. A radiographic nodular-bronchiectasis pattern, sputum AFS titer, radiographic score, and VEGF/PlGF ratio were correlated with NTM-LD status when blinded to data from a second set or later of sputum microbiology for NTM. The predictive index using the four-factor model could be used to screen for NTM-LD. In the clinical application of the predictive index, a score >2 favored the diagnosis of NTM-LD, whereas a score <2 excluded NTM-LD.

NTM-LD and colonization are currently differentiated according to the ATS guidelines. Although the clinical significance of NTM pulmonary colonization is not clear and may be a mild form of lung disease, we investigated ATS-defined NTM-LD in the present study for its greater clinical concern. Compared to the patients with NTM pulmonary colonization, those with NTM-LD had higher levels of circulating VEGF, which has been reported to contribute to an alveolar macrophage response to NTM and the formation of granuloma and inflammation^15^,^17^. In this study, although VEGF was not correlated with radiographic score, the clinical factors that were correlated with VEGF were also all associated with NTM-LD. These included low BMI, high AFS, and high number of positive cultures for NTM, indicating that the level of VEGF indirectly increased with the severity of NTM-LD.

In contrast, a decreased level of circulating PlGF was noted in the patients with NTM-LD. This may have been due to down-regulation by VEGF^22^. In addition, PlGF may induce cell apoptosis of type 2 pneumocytes and play a role in the chronic fibrosis phase. Structural lung diseases such as bronchiectasis and COPD, which involve a higher PlGF level, have also frequently been reported in patients with NTM pulmonary colonization^20^. This may explain the trend of an increase in VEGF/PlGF ratio in the patients with NTM-LD compared to those with NTM colonization. Although the ratio of VEGF/PlGF has not been reported before, this may be useful for discriminating between lung disease of NTM and airway colonization. In particular, the ratio could lead to a bigger difference between NTM-LD and colonization than VEGF or PlGF alone. The ratio was a significant factor in the multivariable logistic regression model and univariable generalized linear model. But it became not significant in multivariable generalized linear model and could be explained by small sample size and the influence from other factors. The real impact of VEGF/PlGF requires further large scale study. For clinical use, the relative risk for NTM-D became smaller and may be more appropriate than the odds ratio which would be over-estimated because the outcome is common^29^.

The VEGF/PlGF ratio was not a good predictor for NTM-LD by itself, and the predictive model included the clinical factors of sputum AFS titer and radiographic findings. Sputum microbiology is one of the most important factors when diagnosing NTM-LD. The AFS titer has been reported to be higher in patients with NTM-LD than in those with NTM pulmonary colonization^14^. After rapidly excluding *M. tuberculosis* in AFS-positive sputum using nucleic acid amplification, the positivity of AFS may be indicative of NTM-LD although AFS titer is not included in the updated NTM-LD guidelines^5^. Previous studies have reported that different NTM species have different levels of clinical relevance, and that this affects the identification of NTM-LD^9^,^10^. However, the NTM species did not affect the prediction of NTM-LD in the present study, which may be because we only recruited patients who were sputum-positive for MAC or MAB species, both of which have a relatively higher clinical relevance than other NTM species^8^.

With regards to radiographic pattern, fibro-cavitary and nodular-bronchiectasis patterns are typically found in patients with NTM-LD^30^,^31^,^32^. However, the pattern is not always specific and it can be biased by other lung diseases such as fungal or *M. tuberculosis* infections^30^,^33^,^34^. In the present study, 33% of the NTM colonization group also had a nodular-bronchiectasis pattern. Specific signs such as feeding bronchus or tree-in-buds may be limited due to the facility of CT^31^,^32^. Thus, combining microbiologic and radiologic factors and biomarkers such as VEGF/PlGF may be a better index for predicting disease status while NTM is being isolated from set of sputum.

This study has several limitations. First, the patients were enrolled from a single medical center, and they had a high rate of underlying co-morbidities, which may have influenced the levels of the biomarkers. Second, more than half of the patients with NTM isolated from sputum were not recruited due to the absence of follow-up. In addition, the control group was enrolled in a different period. Case selection bias may therefore exist. Third, one case in the NTM pulmonary colonization group only had one sputum sample for mycobacterium culture, and this may have led to classification bias. Fourth, CT imaging was not routinely performed, which may have caused interpretation bias in lung imaging.

In conclusion, the rate of NTM-LD was far lower than 100% in the patients with NTM isolated in their sputum. The diagnosis requires multiple factors and can be delayed by the turn-around time of mycobacterial cultures. Before obtaining more than one set of positive sputum cultures for NTM, our proposed predictive index including ratio of circulating VEGF/PlGF, presence of a nodular-bronchiectasis pattern, positive sputum AFS, and score of radiographic extent may be helpful for favoring or excluding NTM-LD in clinical practice.

## Additional Information

**How to cite this article**: Lin, C.-H. *et al*. The trend and the disease prediction of vascular endothelial growth factor and placenta growth factor in nontuberculous mycobacterial lung disease. *Sci. Rep.* **6**, 37266; doi: 10.1038/srep37266 (2016).

**Publisher's note:** Springer Nature remains neutral with regard to jurisdictional claims in published maps and institutional affiliations.

## Supplementary Material

Supplementary Information

## Figures and Tables

**Table 1:** Clinical characteristics of the study participants grouped into nontuberculous mycobacteria (NTM)-lung disease (LD) or colonization.

	NTM-LD (n = 32)	NTM colonization (n = 93)	*p* value
Age, year	63.0 (11.9)	67.4 (13.9)	0.091
Male sex	13 (41%)	41 (44%)	0.733
BMI	20.5 (2.9)	22.3 (3.8)	0.015
Smoking status			
Current smoking	0	5 (5%)	0.181
Ex-smoking	8 (25%)	18 (19%)	0.497
Co-morbidities			
Malignancy	2 (6%)	8 (9%)	0.672
Diabetes mellitus	2 (6%)	9 (10%)	0.555
ESRD	1 (3%)	2 (2%)	0.756
Autoimmune disease	3(10%)	6 (7%)	0.570
COPD	6 (19%)	13 (14%)	0.517
Asthma	2 (6%)	5 (5%)	0.853
Bronchiectasis	15 (47%)	21 (23%)	0.007
Old TB	13 (44%)	24 (26%)	0.113
Radiographic pattern			
Fibro-cavitary	10 (31%)	0	<0.001
Nodular-Bronchiectasis	17 (53%)	31 (33%)	0.047
Radiographic score	3.6 (1.6)	1.9 (2.0)	<0.001
Presence of symptoms¶	31 (97%)	77 (83%)	0.045
Chronic cough	31 (97%)	68 (73%)	0.004
Dyspnea	15 (47%)	27 (29%)	0.065
Hemoptysis	8 (25%)	21 (23%)	0.780
Constitutional symptoms	14 (44%)	25 (27%)	0.075
Symptom duration, days	957 (1334)	669 (1505)	0.388
Mycobacteriology of sputum			
MAC	19 (59%)	68 (73%)	0.145
AFS, titer	1.8 (1.6)	0.2 (0.7)	<0.001
No. of AFS (+) sputum	2.5 (3.1)	0.2 (0.5)	<0.001
No. of NTM (+) sputum	4.1 (2.2)	1.3 (0.6)	<0.001
PlGF, pg/ml	13.4 (18.2)	45.0 (124.0)	0.019
VEGF, pg/ml	24.1 (23.0)	14.7 (14.0)	0.035
VEGF/PlGF	2.7 (2.7)	1.6 (1.9)	0.042

AFS, acid fast smear; COPD, chronic obstructive pulmonary disease; ESRD, end-stage renal disease; MAC, *mycobacterium avium* complex; PlGF, placenta growth factor; TB, tuberculosis; VEGF, vascular endothelial growth factor. Data were presented as number (%) or mean (standard deviation).

^¶^Chronic cough, hemoptysis, dyspnea, and constitutional symptoms.

**Table 2:** Multivariable logistic analysis for predicting nontuberculous mycobacteria (NTM)-lung disease in patients with sputum positive for NTM if without information on the number of positive culture.

Characteristics	Multivariable*	
	*p* value	OR (95% C.I.)
AFS, titer	0.010	2.007 (1.181–3.411)
Nodular-bronchiectasis pattern	0.003	8.580 (2.069–35.575)
Radiographic score	0.089	1.272 (0.964–1.680)
VEGF/PlGF ratio	0.013	1.372 (1.068–1.761)

AFS, acid fast smear; OR, odds ratio; PlGF, placenta growth factor; VEGF, vascular endothelial growth factor.

^*^Multivariable analysis was performed by forward conditional factor selection and the model included age, sex, NTM species, body mass index, radiographic pattern and score, chronic cough, AFS titer, mycobacterial species, plasma PlGF, plasma VEGF, and VEGF/PlGF ratio.

**Table 3:** Relative risk for nontuberculous mycobacteria (NTM)-lung disease in patients with sputum positive for NTM by using generalized linear model with a Poisson regression.

Characteristics	Multivariable*	
	*p* value	Relative Risk (95% C.I.)
AFS, titer, per 1 grade increment	<0.001	1.636 (1.295–2.068)
Nodular-bronchiectasis pattern	0.037	2.212 (1.049–4.651)
Radiographic score	0.038	1.189 (1.010–1.399)
VEGF/PlGF ratio	0.472	1.048 (0.923–1.189)

AFS, acid fast smear; PlGF, placenta growth factor; VEGF, vascular endothelial growth factor.

^*^Multivariable analysis was performed including factors of radiographic pattern and score, AFS titer, and VEGF/PlGF ratio.

**Table 4:** Performance of different combination of predictors for predicting nontuberculous mycobacteria (NTM)-lung disease in patients with sputum positive for NTM.

Markers	Positive* criteria	Sensitivity	Specificity	PPV	NPV	LR+	LR−
AFS titer	>0	59%	88%	78%	77%	4.92	0.47
Radiographic score	>2	81%	73%	51%	92%	3.00	0.26
VEGF/PlGF	>1.8	50%	75%	41%	81%	2.00	0.67
NB pattern	presence	53%	67%	35%	81%	1.61	0.70
Predictive index scoreǂ	>1	94%	75%	56%	97%	3.76	0.08
Predictive index scoreǂ	>2	50%	94%	73%	85%	8.33	0.53
Probability by regression model	>0.21	91%	77%	58%	96%	3.96	0.12

AFS, acid fast smear; LR+, positive likelihood ratio; LR−, negative likelihood ratio; NB, nodular bronchiectasis; NPV, negative predictive value; PlGF, placenta growth factor; PPV, positive predictive value; VEGF, vascular endothelial growth factor.

^*^Was defined by optimal cut-off value according to Youden index method.

^ǂ^Includes index factors of AFS >0, NB radiographic pattern, VEGF/PlGF >1.8, and radiographic score >2. One point was given for every criteria was positive. Then predictive index score was obtained and ranged 0–4.

## References

[b1] Lai C. C. . Increasing incidence of nontuberculous mycobacteria, Taiwan, 2000–2008. Emerg Infect Dis 16, 294–296 (2010). 20113563 10.3201/eid1602.090675 PMC2958002

[b2] Field S. K. & Cowie R. L. Lung disease due to the more common nontuberculous mycobacteria. Chest 129, 1653–1672 (2006). 16778288 10.1378/chest.129.6.1653

[b3] Adjemian J., Olivier K. N., Seitz A. E., Holland S. M. & Prevots D. R. Prevalence of nontuberculous mycobacterial lung disease in US Medicare beneficiaries. Am J Respir Crit Care Med 185, 881–886 (2012). 22312016 10.1164/rccm.201111-2016OC PMC3360574

[b4] Prevots D. R. & Marras T. K. Epidemiology of human pulmonary infection with nontuberculous mycobacteria: a review. Clin Chest Med 36, 13–34 (2015). 25676516 10.1016/j.ccm.2014.10.002 PMC4332564

[b5] Griffith D. E. . An official ATS/IDSA statement: diagnosis, treatment, and prevention of nontuberculous mycobacterial diseases. Am J Respir Crit Care Med 175, 367–416 (2007). 17277290 10.1164/rccm.200604-571ST

[b6] Menzies D. & Nahid P. Update in tuberculosis and nontuberculous mycobacterial disease 2012. Am J Respir Crit Care Med 188, 923–927 (2013). 24127799 10.1164/rccm.201304-0687UP

[b7] Lu D., Heeren B. & Dunne W. M. Comparison of the Automated Mycobacteria Growth Indicator Tube System (BACTEC 960/MGIT) with Lowenstein-Jensen medium for recovery of mycobacteria from clinical specimens. Am J Clin Pathol 118, 542–545 (2002). 12375641 10.1309/65KN-2M7E-7MNN-X0TA

[b8] Koh W. J. . Clinical significance of nontuberculous mycobacteria isolated from respiratory specimens in Korea. Chest 129, 341–348 (2006). 16478850 10.1378/chest.129.2.341

[b9] van Ingen J. . Clinical relevance of non-tuberculous mycobacteria isolated in the Nijmegen-Arnhem region, The Netherlands. Thorax 64, 502–506 (2009). 19213773 10.1136/thx.2008.110957

[b10] Shu C. C. . Nontuberculous mycobacteria pulmonary infection in medical intensive care unit: the incidence, patient characteristics, and clinical significance. Intensive Care Med 34, 2194–2201 (2008). 18648768 10.1007/s00134-008-1221-6

[b11] Ito Y. . Predictors of 5-year mortality in pulmonary Mycobacterium avium-intracellulare complex disease. Int J Tuberc Lung Dis 16, 408–414 (2012). 22230733 10.5588/ijtld.11.0148

[b12] Kitada S. . Long-term radiographic outcome of nodular bronchiectatic Mycobacterium avium complex pulmonary disease. Int J Tuberc Lung Dis 16, 660–664 (2012). 22410245 10.5588/ijtld.11.0534

[b13] Lee M. R. . Factors associated with subsequent nontuberculous mycobacterial lung disease in patients with a single sputum isolate on initial examination. Clinical microbiology and infection: the official publication of the European Society of Clinical Microbiology and Infectious Diseases 21, 250 e251–257 (2015). 10.1016/j.cmi.2014.08.025 25658545

[b14] Shu C. C. . Use of soluble triggering receptor expressed on myeloid cells-1 in non-tuberculous mycobacterial lung disease. Int J Tuberc Lung Dis 15, 1415–1420 (2011). 22283904 10.5588/ijtld.10.0786

[b15] Barleon B. . Migration of human monocytes in response to vascular endothelial growth factor (VEGF) is mediated via the VEGF receptor flt-1. Blood 87, 3336–3343 (1996). 8605350

[b16] Berse B., Brown L. F., Van de Water L., Dvorak H. F. & Senger D. R. Vascular permeability factor (vascular endothelial growth factor) gene is expressed differentially in normal tissues, macrophages, and tumors. Mol Biol Cell 3, 211–220 (1992). 1550962 10.1091/mbc.3.2.211 PMC275520

[b17] Datta M. . Anti-vascular endothelial growth factor treatment normalizes tuberculosis granuloma vasculature and improves small molecule delivery. Proceedings of the National Academy of Sciences of the United States of America 112, 1827–1832 (2015). 25624495 10.1073/pnas.1424563112 PMC4330784

[b18] Alatas F. . Vascular endothelial growth factor levels in active pulmonary tuberculosis. Chest 125, 2156–2159 (2004). 15189936 10.1378/chest.125.6.2156

[b19] Nishigaki Y. . Increased serum level of vascular endothelial growth factor in Mycobacterium avium complex infection. Respirology 11, 407–413 (2006). 16771909 10.1111/j.1440-1843.2006.00863.x

[b20] Cheng S. L., Wang H. C., Yu C. J. & Yang P. C. Increased expression of placenta growth factor in COPD. Thorax 63, 500–506 (2008). 18202163 10.1136/thx.2007.087155 PMC2571977

[b21] Cheng S. L., Wang H. C., Cheng S. J. & Yu C. J. Elevated placenta growth factor predicts pneumonia in patients with chronic obstructive pulmonary disease under inhaled corticosteroids therapy. BMC pulmonary medicine 11, 46 (2011). 21962211 10.1186/1471-2466-11-46 PMC3195784

[b22] Tsao P. N. . Overexpression of placenta growth factor contributes to the pathogenesis of pulmonary emphysema. Am J Respir Crit Care Med 169, 505–511 (2004). 14644931 10.1164/rccm.200306-774OC

[b23] Shu C. C. . Clinical characteristics and prognosis of nontuberculous mycobacterial lung disease with different radiographic patterns. Lung 189, 467–474 (2011). 21956280 10.1007/s00408-011-9321-4

[b24] Hoefsloot W. . The geographic diversity of nontuberculous mycobacteria isolated from pulmonary samples: an NTM-NET collaborative study. Eur Respir J 42, 1604–1613 (2013). 23598956 10.1183/09031936.00149212

[b25] Lin H. H., Ezzati M., Chang H. Y. & Murray M. Association between tobacco smoking and active tuberculosis in Taiwan: prospective cohort study. Am J Respir Crit Care Med 180, 475–480 (2009). 19542475 10.1164/rccm.200904-0549OC

[b26] Snider G. L., Doctor L., Demas T. A. & Shaw A. R. Obstructive airway disease in patients with treated pulmonary tuberculosis. Am Rev Respir Dis 103, 625–640 (1971). 5579906 10.1164/arrd.1971.103.5.625

[b27] Shu C. C. . Hepatotoxicity due to first-line anti-tuberculosis drugs: a five-year experience in a Taiwan medical centre. Int J Tuberc Lung Dis 17, 934–939 (2013). 23743313 10.5588/ijtld.12.0782

[b28] Society A. T. Diagnostic standards and classification of tuberculosis and other mycobacterial diseases. Am Rev Respir Dis 123, 343–358 (1981). 7224347 10.1164/arrd.1981.123.3.343

[b29] Zhang J. & Yu K. F. What’s the relative risk? A method of correcting the odds ratio in cohort studies of common outcomes. JAMA 280, 1690–1691 (1998). 9832001 10.1001/jama.280.19.1690

[b30] Rozenshtein A., Hao F., Starc M. T. & Pearson G. D. Radiographic appearance of pulmonary tuberculosis: dogma disproved. AJR Am J Roentgenol 204, 974–978 (2015). 25905930 10.2214/AJR.14.13483

[b31] Han D. . Radiographic and CT findings of nontuberculous mycobacterial pulmonary infection caused by Mycobacterium abscessus. AJR Am J Roentgenol 181, 513–517 (2003). 12876037 10.2214/ajr.181.2.1810513

[b32] Jeong Y. J. . Nontuberculous mycobacterial pulmonary infection in immunocompetent patients: comparison of thin-section CT and histopathologic findings. Radiology 231, 880–886 (2004). 15118112 10.1148/radiol.2313030833

[b33] Cornillet A. . Comparison of epidemiological, clinical, and biological features of invasive aspergillosis in neutropenic and nonneutropenic patients: a 6-year survey. Clin Infect Dis 43, 577–584 (2006). 16886149 10.1086/505870

[b34] Hwang J. H. . Pulmonary nocardiosis with multiple cavitary nodules in a HIV-negative immunocompromised patient. Intern Med 43, 852–854 (2004). 15497524 10.2169/internalmedicine.43.852

