# Supplementary material for: The trend and the disease prediction of vascular endothelial growth factor and placenta growth factor in nontuberculous mycobacterial lung disease
Source: Sci Rep. 2016 Nov 23;6:37266. doi: 10.1038/srep37266 (PMC5120340; doi:10.1038/srep37266)

**The trend and the disease prediction of vascular endothelial growth factor and placenta growth factor in nontuberculous mycobacterial lung disease**

Chou-Han Lin^1^, Chin-Chung Shu^2,3,4^, Chia-Lin Hsu^4^, Shih-Lung Cheng^1,5^, Jann-Yuan Wang^4^, Chong-Jen Yu^4^, and Li-Na Lee^4,6^

**Correspondence to:**

Chin-Chung Shu, MD

Department of Traumatology

National Taiwan University Hospital

# 7, Chung-Shan South Road, Taipei 100, Taiwan

E-mail: ccshu139@ntu.edu.tw

Tel: 886-2-23562905; Fax: 886-2-23582867

and

Cheng Shih-Lung, MD, PhD

Department of Internal Medicine

Far-Eastern Memorial Hospital, New Taipei City, Taiwan and

Department of Chemical Engineering and Materials Science,

Yuan Ze University, Zhongli City, Taoyuan County

E-mail: chest5415@hotmail.com

**Running title:** VEGF and PlGF in NTM lung disease

**Conflict of interest statement:** None

___________________________

^1^Department of Internal Medicine, Far-Eastern Memorial Hospital, New Taipei City, Taiwan

^2^Graduate Institute of Clinical Medicine, College of Medicine, National Taiwan University, Taipei, Taiwan

^3^Department of Traumatology, ^4^Department of Internal Medicine, and ^6^Department of Laboratory Medicine, National Taiwan University Hospital, Taipei, Taiwan

^5^Department of Chemical Engineering and Materials Science, Yuan Ze University, Zhongli City, Taoyuan County

**Online Supplement File**

1. The equation shown below was used to calculate the probability of NTM-LD in the multivariable logistic regression model.

**Equation**: *Logit* (Probability) = 0.241 x (chest radiographic score) + 0.697 x AFS titer + 0.316 x VEGF/PlGF + 2.149 x presence of NB pattern* - 4.478.

*presence of NB pattern = 1, and absence = 0

NB, nodular bronchiectatic radiographic pattern; AFS, acid fast smear

1. Chest radiographic scoring: In brief, we divided each lung field into three zones according to two horizontal lines located at the distal end of the lobar pulmonary artery (Figure E1). We rated each zone from 0 to 3 points. If a lesion involved equal to or less than one-third of the area, we gave it one point. If a lesion involved more than one-third but less than two-thirds of the area, we gave it two points.

Figure E1. Example of the area of chest radiographic score


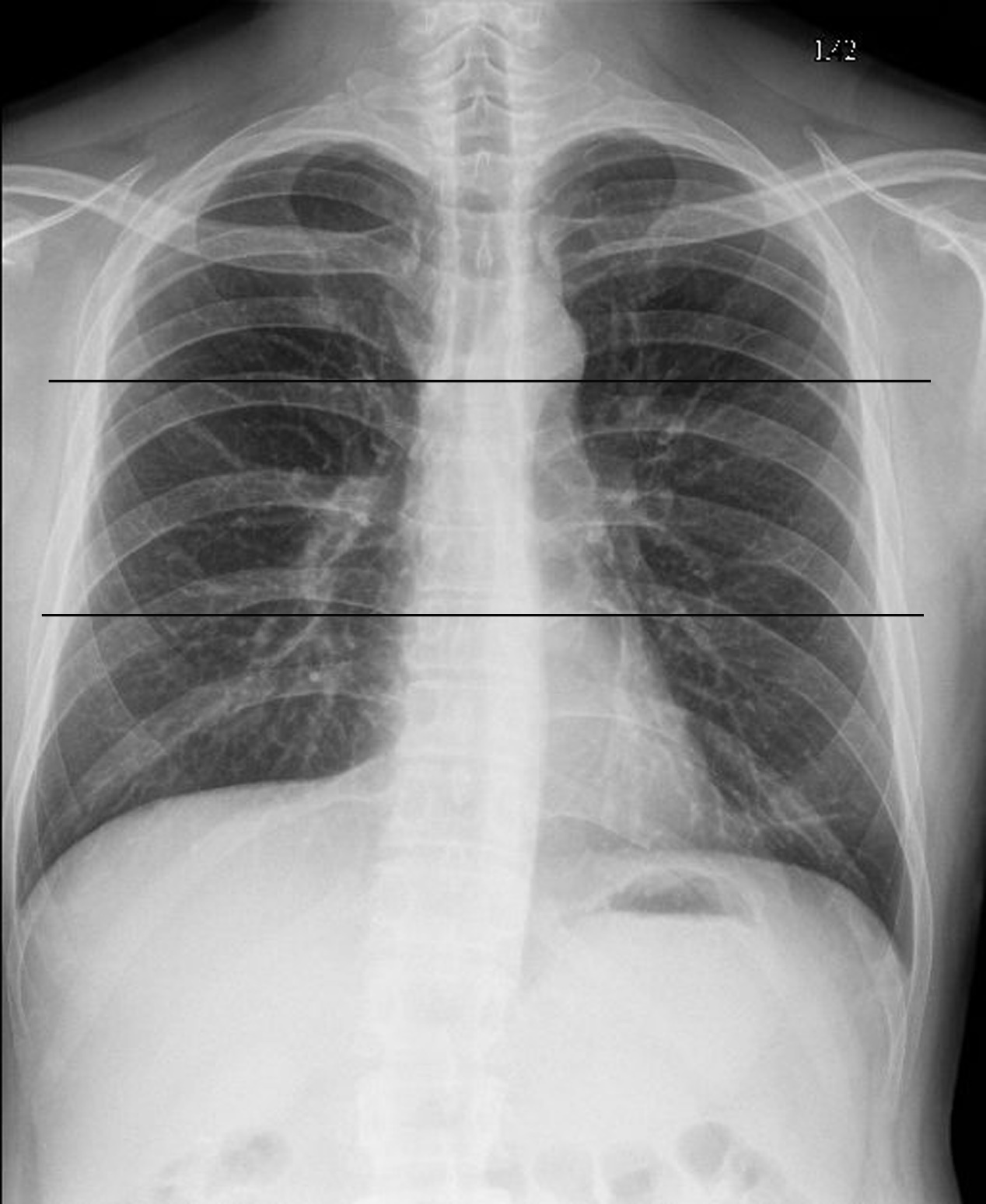

Supplement: Supplementary Information [file srep37266-s1.doc]
